# PSMA-Targeting Radiopharmaceuticals for Prostate Cancer Therapy: Recent Developments and Future Perspectives

**DOI:** 10.3390/cancers13163967

**Published:** 2021-08-05

**Authors:** Mohamed El Fakiri, Nicolas M. Geis, Nawal Ayada, Matthias Eder, Ann-Christin Eder

**Affiliations:** 1 Department of Nuclear Medicine, University Medical Center Freiburg, Faculty of Medicine, University of Freiburg, Hugstetter Str. 55, 79106 Freiburg, Germany; mohamed.el.fakiri@uniklinik-freiburg.de (M.E.F.); nicolas.geis@uniklinik-freiburg.de (N.M.G.); nawal.ayada.amgar@uniklinik-freiburg.de (N.A.); ann-christin.eder@uniklinik-freiburg.de (A.-C.E.); 2 Division of Radiopharmaceutical Development, German Cancer Consortium (DKTK), Partner Site Freiburg, and German Cancer Research Center (DKFZ), 69120 Heidelberg, Germany; 3 Faculty of Biology, University of Freiburg, 79104 Freiburg, Germany

**Keywords:** PSMA, prostate-specific membrane antigen, targeted radionuclide therapy, prostate cancer, theranostics

## Abstract

**Simple Summary:**

One of the most frequently diagnosed cancer in men is adenocarcinoma of the prostate. Once the disease is metastatic, only very limited treatment options are available, resulting in a very short median survival time of 13 months; however, this reality is gradually changing due to the discovery of prostate-specific membrane antigen (PSMA), a protein that is present in cancerous prostate tissue. Researchers have developed pharmaceuticals specific for PSMA, ranging from antibodies (mAb) to low-molecular weight molecules coupled to beta minus and alpha-emitting radionuclides for their use in targeted radionuclide therapy (TRT). TRT offers the possibility of selectively removing cancer tissue via the emission of radiation or radioactive particles within the tumour. In this article, the major milestones in PSMA ligand research and the therapeutic developments are summarised, together with a future perspective on the enhancement of current therapeutic approaches.

**Abstract:**

Prostate cancer (PC) is the second most common cancer among men, with 1.3 million yearly cases worldwide. Among those cancer-afflicted men, 30% will develop metastases and some will progress into metastatic castration-resistant prostate cancer (mCRPC), which is associated with a poor prognosis and median survival time that ranges from nine to 13 months. Nevertheless, the discovery of prostate specific membrane antigen (PSMA), a marker overexpressed in the majority of prostatic cancerous tissue, revolutionised PC care. Ever since, PSMA-targeted radionuclide therapy has gained remarkable international visibility in translational oncology. Furthermore, on first clinical application, it has shown significant influence on therapeutic management and patient care in metastatic and hormone-refractory prostate cancer, a disease that previously had remained immedicable. In this article, we provide a general overview of the main milestones in the development of ligands for PSMA-targeted radionuclide therapy, ranging from the firstly developed monoclonal antibodies to the current state-of-the-art low molecular weight entities conjugated with various radionuclides, as well as potential future efforts related to PSMA-targeted radionuclide therapy.

## 1. Introduction

Prostate cancer (PC) is one of the most commonly diagnosed malignancies among men. PC prevalence is variable throughout the world, being the second most common cancer in men in Europe and the USA, while it is much less prevalent in eastern Asia, North Africa and the Middle East [1]. In 2020, almost 1.4 million men worldwide suffered from PC. Moreover, estimations indicate a gradual increase in PC-affected men worldwide, with 2.3 million cases predicted in 2040 [2]. PC is often asymptomatic, without any specific need for treatment, although a significant percentage (30%) of patients develop metastases of different severity that can lead to a morbid state and even death [3].

Specifically, metastatic castration-resistant prostate cancer (mCRPC) is a fatal form of prostate cancer with widespread metastases in the body, particularly in the bones [4]. As of today, the main burden in the treatment of mCRPC is the difficulty to completely eradicate the cancerous cells [5]. Nevertheless, notable progress in the treatment of mCRPC has been attained over recent years with targeted radionuclide therapy (TRT) approaches [6]. TRT takes advantage of cytotoxic, highly energetic radioactive nuclides, which are conjugated to high-affinity ligands targeting overexpressed markers in cancerous tissue, thereby selectively removing tumours while sparing healthy tissue [7]. Moreover, theranostics represent a promising approach in the field of nuclear medicine, referring to compounds that can be used for both therapy and imaging [8]. Some radiopharmaceuticals (e.g., PSMA-617) are typical examples, as they can be complexed with positron-emitters (e.g., ^68^Ga, ^44^Sc or ^152^Tb) for imaging and with the beta minus (β^-^) ^177^Lu or alpha (α) ^225^Ac-emitters for the treatment of the previously staged lesions [9,10,11]. As a consequence, theranostic compounds facilitate clinical applications and allow for personalised and precise treatment.

**Figure 1:**
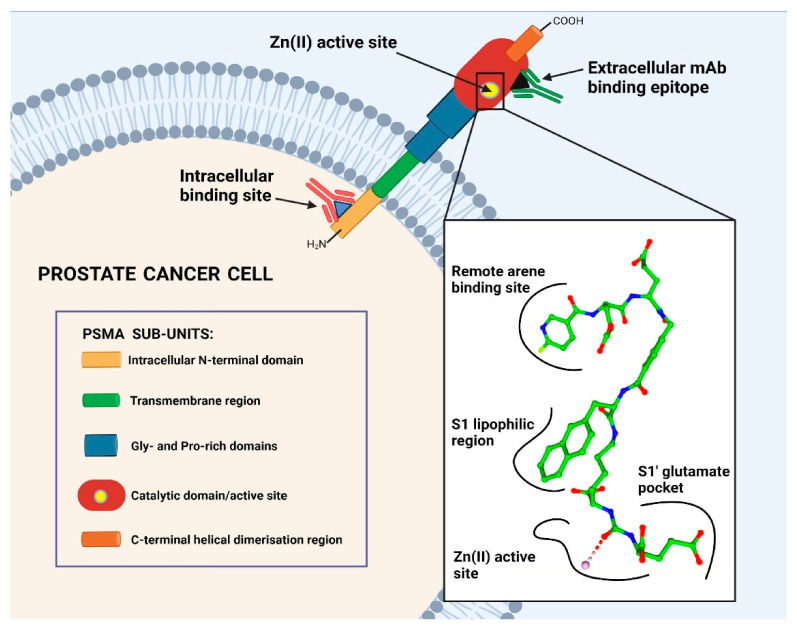
Schematic drawing of PSMA in the cell membrane. The various domains are represented along with the intracellular and extracellular mAb-binding epitopes. Moreover, a schematic drawing of PSMA-1007 in complex with the binding funnel is displayed with the main features present in the catalytic site of PSMA.

In particular, for mCRPC, PSMA represents the ideal target for TRT approaches as it is physiologically expressed in prostatic tissue, with 100 to 1000 times upregulated expression in mCRPC cells as compared to healthy cells [12]. Several hypotheses have been formulated with respect to the role of PSMA in the prostate and in PC progression, although it remains unknown thus far [13]; however, the physiological role of PSMA in other tissues is well known. PSMA is a type II transmembrane glycoprotein characterised by three main segments: a short N-terminal intracellular section, a transmembrane domain and a large extracellular section (C-terminus) (Figure 1). The extracellular segment is the most important with regard to the enzymatic function of this glycoprotein, as it contains the catalytic domain and a zinc-containing, substrate-binding site capable of interacting with specific inhibitors [14]. The enzymatic binding site of PSMA is organised as a pocket with a funnel located at the entrance. In the active centre, PSMA inhibitors interact tightly with zinc ions and charged amino acids, whereas the funnel accepts rather lipophilic interactions. In Figure 1, a typical PSMA inhibitor is shown, addressing all necessary interactions for tight binding with the active centre and the hydrophobic funnel. Compounds interacting well with this pocket show the potential to internalise upon binding. After internalisation, low-molecular weight inhibitors were shown to be released in the cytoplasm while the receptor was recycled [15]. The accumulation of the inhibitor inside the cell results in excellent tumour retention, which is ideal for imaging and therapy.

PSMA has been successfully targeted by many entities, ranging from monoclonal antibodies (mAbs) to low-molecular weight (LMW) peptidomimetics, proving the feasibility of PSMA-TRT as a powerful tool in the management of severe mCRPC [6]. This review does not intend to be systematic but solely presents the main milestones in PSMA-targeted research, from the conception of the first mAbs, to the development of the current ‘gold standard’ PSMA-617, while emphasising the differential properties of biomolecules and LMW peptidomimetics. Moreover, primary β- and α-based therapeutic studies will be described, along with radionuclide comparisons and an outlook on the recent challenges and future of PSMA-TRT.

## 2. PSMA-Targeting Molecules: From mAbs to LMW Peptidomimetics

### 2.1. Monoclonal Antibodies (mAbs) Targeting PSMA

Over the last decades, notable efforts have been made to design and optimise PSMA-targeting compounds for imaging and therapy of PC. The first designed PSMA-binding entities for nuclear medicine were monoclonal antibodies (mAbs). In 1987, Horoszewicz et al. developed 7E11, a murine-derived mAb that specifically binds to PSMA(+) cells [16]. Subsequently, 7E11 was conjugated to pendetide, forming the immunoconjugate known as CYT-356 [17]. Pharmacokinetic and immunohistochemical studies on CYT-356 revealed specific reactivity towards PSMA. Furthermore, in vivo mice biodistribution studies showed good results, with up to 30% of the injected dose of mAb accumulating in tumourous tissue after 3 days, without accumulation in off-target organs, as well as sufficient tumour-to-blood ratios [17]. Taking into account the satisfactory profile shown by 7E11/CYT-356 in preclinical models, this was translated into clinical applications. In this respect, studies carried out by Babaian et al. demonstrated that an ^111^In-labelled version of 7E11 could be employed in imaging pelvic lymph node metastases, with an overall sensitivity of 76% [18]. Nevertheless, 7E11 is limited to soft tissue tumourous lesions due to its inherent binding properties. The mAb binds to an intracellular epitope of PSMA and must transfuse into the tumour via non-specific pathways (e.g., permeable foci of dead cells due to tumour overgrowth). This makes 7E11 less sensitive to bone or compact tissue metastases, which limits its applicability in theranostic approaches [19].

The need of intracellular PSMA-targeting mAbs to internalise via non-specific pathways impede their widespread application in systematic approaches. Consequently, efforts have been and are still being made to develop extracellular PSMA-targeting mAbs, aiming to overcome the main burdens of intracellular targeting mAbs [19]. In this regard, Bander et al. reported the first extracellular PSMA-binding mAbs comprising a series of mAbs (J591, J533, J415 and E99), which were evaluated in vitro by Smith-Jones et al. [16]. For their evaluation, the mAbs were conjugated with a DOTA-chelator for ^111^In labelling or directly labelled with ^131^I. In a competitive binding assay towards PSMA(+) LNCaP cells, all mAbs showed nanomolar affinities, with J591 displaying the highest binding affinity (K_d_ = 1.83 ± 1.21 nmol/L). Furthermore, after binding, the mAb–PSMA complexes were rapidly internalised by LNCaP cells via endocytosis. With these results in hand, the same team of researchers pursued preclinical evaluation of the mAbs in LNCaP tumour-bearing mice; the study corroborated the in vitro findings. The mAbs were PSMA-specific, showing no binding towards PSMA(-) cell lines. Biodistribution of the extracellular mAbs was comparable to the intracellular 7E11, with long circulation times and peak tumour accumulation 4 days p.i. (15.7 ± 3.5%ID/g for [^111^In]In-J591). Moreover, the extracellular mAbs, specifically [^111^In]In-J591, were remarkably cleared from the bloodstream significantly faster than 7E11 [20,21,22]. The faster clearance of J591 led to improved tumour-to-blood ratios and provided the rationale for clinical mAb-based PSMA-TRT.

Consequently, the murine-derived J591 was humanised (huJ591) to avoid adverse immune responses from repeated murine-based mAb dosing [23]. After first-in-human imaging studies with [^89^Zr]Zr-huJ591, the mAb caused satisfactory tumour-to-background ratios 8 days p.i. and 95% imaging accuracy of bone metastases [24]. The mAb was radiolabelled with the therapeutic radionuclides ^90^Y and ^177^Lu and administered in phase I/II trials. Nevertheless, in spite of the relatively good biodistribution and pharmacokinetic profiles of huJ591, only 8% of patients receiving [^177^Lu]Lu-huJ591 showed significant disease remission, with a time-sustained decline in PSA levels ≥ 50% [25].

In addition to J591, other second-generation extracellular PSMA-targeting mAbs have been developed, such as 3/F11, 3C6 and 3/E7. These were tested in preclinical PET imaging studies, showing typical pharmacokinetic profiles of mAbs, namely long circulation times and good tumour accumulation and tumour-to-blood ratios after a few days [26,27]. Third-generation mAbs have also been developed in conjugation with fluorescent agents for hybrid approaches, such as the IgGD2B, which was recently developed, radiolabelled with ^111^In and tagged with the fluorescent moiety IRDye800CW [28,29].

Nevertheless, the reported characteristics and properties of mAbs generally limit their application in theranostic approaches. Their large size (~150,000 Da) prolongs their circulation times in comparison to LMW ligands. Furthermore, they show reduced penetration in tissue, making it more difficult for mAbs to reach their target. Additionally, in a similar way to endogenous substrates, mAbs are metabolised to their constituting amino acids by proteases or peptidases and are further excreted through the bile, faeces or urine (for the smallest fragments). The long circulation times along with the slow penetration characteristics give mAbs extended half-life in the bloodstream, delivering an undesired dose of radiation to healthy tissue. In a phase I/II imaging study with ^89^Zr-labelled J591, it was concluded that mAbs have some practical limitations due to their size. Nearly 8 days were required for high-contrast imaging and the high background noise at early time points interfered with the detection of certain single lesions. Given these limitations, the authors of this prospective study concluded that low molecular weight agents with more rapid clearance profiles might be more suitable for nuclear medicine applications [24]. Nevertheless, mAbs are generally highly specific for their target, causing very minor off-target effects and usually low toxicities. Imaging studies with [^89^Zr]Zr-J591 antibody have demonstrated accumulation primarily by the liver, minimal uptake in the urinary tract and no uptake in the salivary or lacrimal glands. During [^177^Lu]Lu-J591 therapy, the primary reported dose-limiting side effect of the radiolabelled antibody was myelosuppression, a typical event of long circulating compounds causing an excessive undesired radiation burden to the bone marrow [30]. Although rare, mAbs can interfere with vital processes and lead to severe adverse reactions, such as cytokine release syndrome or encephalopathy [31].

Due to the reported limitations of mAbs, researchers have shortened the length and weight of the biomolecules in order to reduce their circulation time and allow for generally faster accumulation or penetration in tumourous tissues. Following this line of reasoning, the first PSMA-targeting minibodies or diabodies were derived from huJ591 single-chain fragments (scFv). In a study reported by Viola-Villegas et al., two different mAb derivatives were obtained from huJ591: Mb (~80 kDa minibody) and Db (~40 kDa diabody). Both entities were radiolabelled with ^89^Zr to assess their biodistribution profiles in mice bearing LNCaP xenografts. Their pharmacokinetic profile outperformed the parent mAb at early time points, with maximal tumour accumulation 24 h p.i. for [^89^Zr]Zr-Mb (12.1 ± 3.6% ID/g) and 12 h p.i. for [^89^Zr]Zr-Db (12.3 ± 2.5% ID/g). Furthermore, both fragments showed comparable specificities towards PSMA when compared with their parent mAb [32].

Subsequently, even smaller mAb-derived molecules were developed, e.g., nanobodies, which are the smallest mAb derivatives, with high affinity and specificity towards their target [33]. Moreover, due to their small size, they reach epitopes that are prohibited for full mAbs [34]. Some of the first nanobodies developed for PSMA were reported by Evazalipour et al. A series of nanobodies were labelled with ^99m^Tc and showed better pharmacokinetic profiles at early timepoints when compared to mAbs. They had high specificity towards PSMA, good internalisation ratios (~16%) and fast renal clearance [35]. Similarly, in 2015, Chatalic et al. developed two nanobodies: JVZ007-c-*myc*-his and JVZ007-cys. The ^111^In-labelled version of the nanobodies showed maximal tumour accumulation 4 h p.i. (3.91 ± 1.13%ID/g and 3.70 ± 0.29%ID/g, respectively) and prohibitive kidney uptake that could only be reduced by co-infusion of gelofusine and lysine [36].

Overall, comprehensive efforts have been made in the development of PSMA-specific mAbs, although ultimately the slow pharmacokinetic profile, which implies long circulation times, limits their application in theranostic concepts. Their properties require the use of long-lived isotopes that release an undesirable radiation burden to healthy tissue. Even though efforts have been realised in shortening the circulation times of mAb-based PSMA ligands, e.g., by truncation of their chains, the molecular format with the optimal toxicity–efficacy balance in TRT is still to be determined.

### 2.2. Low-Molecular Weight (LMW) PSMA Inhibitors

Low-molecular weight (LMW) molecules targeting PSMA are typically based on a skeleton containing a specific PSMA-binding entity, a linker and a chelator for labelling with radiometals. This ligand–linker–chelator design allows discrete modifications in all three structural elements, with potentially significant impacts on the affinity, pharmacokinetics and pharmacodynamics of the molecule.

With the early discovery of PSMA being identical to NAALADase (N-acetylaspartylglutamate peptidase) [37,38] and folate hydrolase 1 (FOLH-1) [39], the development of LMW molecules targeting PSMA began to gain significance. Previously discovered and already known inhibitors, which were mainly designed for the homologous enzymes, formed the basis for further scientific studies.

#### 2.2.1. Binding Entity

The effective targeting of tumourous cells is one of the main characteristics of cancer-specific (radio)pharmaceuticals, rendering the binding affinity a crucial characteristic for therapeutic success. Furthermore, in oncological radiopharmacy, it is preferable to deposit the radioactivity in the cytoplasm of the malignant cell, thereby emphasising the importance of effective internalisation. Compounds targeting PSMA typically mimic either the natural ligand NAAG (N-acetylaspartylglutamate) or the transition state of the PSMA-catalysed hydrolysis of NAAG; therefore, binding entities of LMW inhibitors targeting PSMA can be divided into three subgroups: phosphorus-based, thiol-based and urea-based structures.

**Figure 2:**
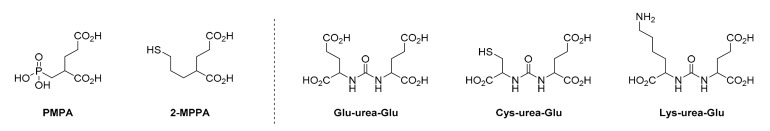
Structures of PSMA-binding entities: (**left**) the initially developed phosphorous-based PMPA and thiol-based 2-MPPA; (**right**) urea-based entities, including the most successfully applied entity, Lys–urea–Glu. All entities comprise a Zn(II) coordinating moiety and glutamic acid.

In 1996, phosphorus-based PSMA inhibitors were introduced by Jackson et al. [40]. The main rationale for these inhibitors was to impede NAALADase function in the context of neurological diseases. As a starting point, the substrate 2-phosphonomethyl pentanedioic acid (2-PMPA) was employed, which showed an inhibition constant (K_i_) of 0.275 ± 0.08 nM and is currently one of the most potent and widely used inhibitors for blocking PSMA (Figure 2) [41]. Based on the high affinity of 2-PMPA, a ^18^F-labelled version (BAY 1075553) was synthesised [42]; however, subsequent investigations in a phase I clinical study with a small cohort of patients produced inferior results in comparison to [^18^F]fluoroethylcholine [43]. Further development included the introduction of substances with a phosphinic or phosphonate core by Jackson et al. [44] and the development of phosphoramidate compounds by Berkman et al. [45,46], with promising results for clinical applications; therefore, the phosphoamidate-derived inhibitor was used in the first phosphorous-based PSMA-targeting LMW molecule for ^18^F-PET imaging [47]. Additionally, in the development of further tracers of this class, the described binding entity served as a basic structure for numerous new radiopharmaceuticals [48,49,50,51,52,53]. With promising results in a phase I clinical study, the PSMA tracer CTT1057 showed great potential as a novel candidate for further clinical testing [53]. Nevertheless, although phosphorus-based PSMA inhibitors play a subordinate role in today’s PSMA-targeted diagnostics and treatment of prostate cancer, ground-breaking work with phosphorus-containing inhibitors served as the starting point for the development of current state-of-the-art molecules.

The first approaches to developing thiol-based PSMA-binding entities were reported by Tsukamotos et al. [54,55]. In this regard, the interaction of the free thiol with the Zn^2+^ binding site of PSMA was investigated in preclinical settings, highlighting 2-MPPA, which showed the most promising results, with an IC_50_ of 90 ± 26 nM and a high concentration in the blood after oral administration (Figure 2) [54]. Further clinical studies with 2-MPPA showed degradation of the chemical structure in healthy subjects and gastrointestinal side effects [56]. With the introduction of aromatic substructures, Stoermer et al. improved the affinity of thiol-based inhibitors to a low nanomolar range [57]. In another study by Ferraris et al., δ-thiolacetone derivatives showed great potential as pro-drugs with high oral bioavailability [58]. Nevertheless, thiol-based PSMA inhibitors currently only play a minor role in the diagnosis and treatment of PC, mainly due to their poor stability and pronounced side effects.

Compared to the aforementioned binding entities, urea-based inhibitors showed significantly better pharmacokinetic properties towards PSMA [59]; therefore, the majority of newly developed LMW PSMA-targeting pharmaceuticals are based upon the Zn^2+^-targeting substructure [60,61]. Inspired by the phosphorous-containing predecessors, urea-bearing structural analogues were first described by Jackson et al. [62]. The simplified synthesis via in situ activation of the glutamic acid moiety through an isocyanate intermediate was a major advance and is widely applied as of today in the manufacture of PSMA-targeting molecules [62]. Furthermore, it was demonstrated that the stereochemistry of glutamic acid is essential for achieving high affinity towards PSMA, resulting in the (*S*)-configuration of the amino acid being the most favourable [62]. As a consequence, the substitution or modification of the glutamic acid in the binding moiety results in a considerable loss of affinity towards PSMA [63,64]. Foss et al. evaluated the potential of urea-based inhibitors in a PSMA-expressing LNCaP mouse xenograft in one of the first in vivo studies [65]. Up to this point, most PSMA-targeting LMW molecules of this class had comprised either a Glu–urea–Glu or Cys–urea–Glu binding entity (Figure 2). Thereafter, the development of the Glu–urea–Lys (EUK) entity introduced a third subgroup of urea-based inhibitors (Figure 2) [66,67]. EUK-based inhibitors have thus far been the base for the most successful PSMA-targeting LMW inhibitors, such as ^18^F-DCFPyL or the FDA-approved, ^68^Ga-labelled PSMA-11 for PET imaging or the theranostic variants PSMA-617 and PSMA I&T. Moreover, scientists recently explored further modifications in this PSMA-binding moiety. For example, Kim et al. demonstrated the negative impacts on affinity towards PSMA when employing β- and γ-amino acids compared to the α-amino acid counterparts [68]. Similar negative effects were observed by Kwon et al. by inverting the configuration of the α-amino acid in the binding region of the moiety [69]. Other efforts related to the design of binding entities were based on sulfamides [70], carbamates [71,72], thioureas [64,73] and dimeric peptides [74,75]. These approaches were generally not extensively evaluated due to the low PSMA-binding affinities.

#### 2.2.2. Linkers and Chelators

In recent years, research on LMW PSMA-targeting molecules for TRT shifted to modifications on the chelator, and especially the linker regions on EUK-based inhibitors. Increasing evidence suggests that the binding affinity and internalisation ratios are not only induced by the PSMA-binding entity, but are also strongly influenced by the moieties present in the linker. Variations of the linker and chelator regions have significant impacts on the pharmacokinetics, pharmacodynamics and biodistribution profiles of PSMA-targeting LMW inhibitors. The early study by Barinka et al. showed the potential to increase the PSMA-binding affinity using short-length linkers and non-polar functionalities targeted towards the entrance region of the PSMA-binding funnel [67]. Further investigations uncovered the interactions of potent PSMA inhibitors with the Zn^2+^ ions and basic amino acids in the PSMA catalytic subpocket, and also lipophilic and π-cationic interactions in the S1 lipophilic region (Figure 1). Furthermore, Zhang et al. reported an additional arene-binding site with potential aromatic stacking interactions of LMW inhibitors [76].

Theranostic PSMA-targeting approaches for diagnosis and treatment of mCRPC have been closely related to the development of highly potent inhibitors. In early PSMA-TRT trials, nuclides emitting α- or β^-^-radiation were introduced by simple nucleophilic substitution on aromatic ring systems bound to urea-based binding entities [77,78].

Two of the first PSMA-targeting small-molecule inhibitors for radionuclide therapy of PC were radio-iodinated MIP-1072 and MIP-1095, as reported by Barrett et al. [79]. Preclinical imaging studies were used to compare the compounds to assess their feasibility for a therapeutical application. The presence of an additional urea group in MIP-1095 was expected to be associated with a higher potency compared to the amine group in MIP-1072, due to increased lipophilicity [80]. Despite both agents clearing rapidly from the blood pool, MIP-1072 showed faster kidney clearance, which was possibly caused by the structural differences [79]. Nevertheless, due to the high tumour uptake of the radiopharmaceutical, [^131^I]I-MIP-1095 was the radiopharmaceutical of choice for further clinical trials [77].

Kiess et al. transferred findings from diagnostic studies to the development of therapeutic variants by synthesizing small molecules based on the EUK binding motive and *para*-substituted benzoic acid, giving rise to the first astatine-bearing LMW inhibitor, [^211^At]At-DCAtBzL [78]. Both iodine and astatine variants of the inhibitors showed comparable uptake into PSMA-expressing cells, demonstrating the potential of iodine compounds as powerful tools to surrogate astatine in preclinical settings; however, the compound was severely hampered by their high kidney uptake. To address this shortcoming, Childers et al. evaluated constitutional isomers of the described inhibitors [81]. While maintaining the lengths of the linker and functional subunits, structural analogues based on a Glu–urea–Glu binding entity showed an 8-fold improvement in the tumour-to-kidney ratio in mice bearing PSMA(+) tumours 21 h p.i. [81]. Recently, Vaidyanathan et al. demonstrated further improvements in the biodistribution and pharmacokinetic profile by adding a guanidino group to the aromatic ring of the inhibitor [82]. In addition, modifications with quinolone derivatives appear advantageous in diagnostic tracers and could potentially serve as templates for future therapeutic approaches [83].

In contrast to previously discussed radionuclides such as ^131^I or ^211^At, radiometals require a complexing agent (chelator) for their introduction to PSMA-targeting inhibitors. The most commonly used chelators for therapeutic approaches are DOTA and DOTAGA, which function as complexing agents for therapeutically relevant trivalent cations such as [^177^Lu]Lu^3+^ or [^225^Ac]Ac^3+^. As an example, in recent years, the DOTA-based PSMA-617 and DOTAGA-bearing PSMA I&T gained dominant positions as ‘gold-standard’ LMW peptidomimetics in TRT of mCRPC.

In order to render PSMA inhibitors suitable for radiometal-based therapy, appropriate chelators have to be introduced into the chemical structures of LMW inhibitors. In 2010, Banerjee et al. reported the first DOTA-based PSMA-targeting inhibitor with a EUK binding entity [84]. The investigated compounds shared the common structural elements of suberic acid and *L*-lysine in the linker region. The addition of two *L*-phenylalanine units next to the DOTA chelator, while preserving the main linker elements, showed improved target-to-tissue ratios with tumour uptakes comparable to the parental structure. In further studies, additional modifications of the linker and chelator regions led to further improvements [85]. Maintaining the EUK unit, a linker consisting of 6-aminohexanoic acid was used to connect the binding motive with HBED-CC (diagnostic) and DOTA (therapeutic) as chelators. While the DOTA derivative showed comparable performance to the inhibitors previously described by Banerjee et al., the HBED-CC conjugate (PSMA-11) provided a significantly improved internalisation in LNCaP cells, resulting in a higher tumour uptake of 7.7%ID/g 1 h p.i. Although PSMA-11 was not a feasible agent for therapeutic approaches, it was still implemented as a diagnostic tracer, with great clinical impact and recent FDA approval [86,87]. In addition, relevant subsequent studies in relation to linker modifications of LMW urea-based PSMA inhibitors made major contributions to the entire field of PSMA theranostics [88,89,90,91].

In 2015, the preclinical development of PSMA-I&T and PSMA-617 was reported and both radiopharmaceuticals were suggested for TRT [92,93]. Both compounds shared the main characteristics of being urea-based, having a chelator able to host trivalent radionuclides and an outstanding pharmacokinetic profile.

In a preliminary study, Weineisen et al. demonstrated the benefits of introducing DOTAGA as a chelator for TRT [94]. While maintaining the previously described linker consisting of suberic acid, lysine and two *L*-phenylalanine building blocks, the DOTAGA conjugate showed significantly improved tumour uptake when compared to the DOTA equivalent and similar performance compared to PSMA-11. By using *L*-amino acids in the linker region, a rapid proteolytic cleavage of the radiolabelled inhibitor was observed. This issue was addressed by substituting the *L*-amino acids by the *D*-amino acid counterparts, thereby improving the in vivo stability and further enhancing the pharmacokinetic profile. In following studies, further optimisation of the linker region led to the third-generation tracer PSMA-I&T. Exploiting the potential of the peptidomimetic linker unit, one *D*-phenylalanine was surrogated by 3-iodo-*D*-tyrosine to increase the lipophilic interaction of the molecule with the remote arene binding site in the PSMA-binding pocket. This modification led to a higher affinity and internalisation ratios towards PSMA and a comparable biodistribution to PSMA-11 [93].

Regarding DOTA-containing inhibitors, a novel approach with new tailormade modifications of the linker region led to an optimised inhibitor for TRT, PSMA-617 [92,95]. Since the simple replacement of HBED-CC in PSMA-11 by DOTA resulted in a significant decrease of the tumour targeting properties, systematic linker alterations were carried out to improve the interaction of the inhibitor with the binding funnel of PSMA [95]. The first set of compounds included different units and arrangements of aromatic rings in the linker region, demonstrating the importance of aromatic moieties inserted between the EUK entity and DOTA. While the compound with three aromatic rings in the linker region showed the most favourable affinity to PSMA, internalisation properties were diminished. In a second set, it was observed that at least one aromatic moiety with a rigid conformation in the linker region is required to preserve affinity, while having sufficient internalisation ratios. Among the investigated substances, PSMA-617 was deemed the best performing, with a linker containing 2-naphthyl-*L*-alanine (2Nal) and trans-4-(aminomethyl) cyclohexanecarboxylic acid (AMCHA). Further modifications showed that both the change in chirality of 2Nal and the modification in constitutional isomerism did not have a positive effect on the pharmacokinetic profile of the molecule. In the series of inhibitors reported by Benesova et al., the only other structural modification with potential comprised a substitution of the cyclohexyl ring by a phenyl. This change resulted in higher affinity towards PSMA but also diminished the clearance ratio from the kidneys when compared to PSMA-617 [95].

In other studies based on PSMA-617, ^68^Ga-labeled derivatives did not show significant improvements when the 2Nal moiety was substituted by 2-indanylglycine (Igl) or 3,3-diphenylalanine (Dip) [96]. Additionally, Wüstemann et al. studied the effects of different chelators on the pharmacological profile while maintaining the core structure of PSMA-617 [97]. Of the eight chelators studied, the inhibitor containing CHX-A″-DTPA showed the most promising results with respect to tumour uptake and retention, although with adverse effects on renal excretion and clearance.

#### 2.2.3. Pharmacokinetic Modifications: Albumin Binders, Charged Spacers and Cleavable Linkers

The addition of different substructures to PSMA-targeting LMW inhibitors has been reported to modify pharmacological characteristics or expand the scope of possible applications. Accordingly, multiple strategies have been exploited in attempts to improve the biodistribution and excretion profiles of current LMW-PSMA inhibitors.

The attachment of albumin binders follows the widely recognised principle that serum protein binding of pharmaceuticals can improve the tumour uptake of otherwise rapidly cleared molecules by expanding their circulation half-life [98]. Extensive studies have been reported related to LMW albumin-binding PSMA inhibitors, such as the initially reported series of inhibitors by Kelly et al. [99] or the more recent Evans-Blue-modified PSMA-617 derivative, which is currently in phase I trials [100,101]. Other remarkable efforts related to albumin-binding PSMA inhibitors were reported by Benesova et al. [102], Umbricht et al. [103], Kuo and co-workers [104,105] and more recently Deberle et al. [106]. In all instances, the tumour uptake of the modified radiopharmaceutical was higher than for PSMA-617, although the longer circulation times also implied higher absorbed doses to non-target tissue.

Additionally, the addition of charged moieties in the linker region [88,89] and the introduction of cleavable linkers [107,108] are strategies that have proven impacts on the pharmacokinetic profiles of PSMA inhibitors. Both modifications result in more accelerated excretion profiles with better tumour-to-organ ratios and lower radiation burden to non-target healthy tissue.

More detailed studies reviewing the main findings related to pharmacokinetic modifications of PSMA inhibitors have been published elsewhere [109,110]. Due to the lack of clinical data, it is currently unclear which role the albumin-binding inhibitors will have in the field of PSMA-TRT. Nevertheless, the introduction of albumin-binding moieties is a simple way to enhance tumour enrichment and has the potential to increase therapeutic efficacies; however, the potential increase in background uptake has to be considered and carefully evaluated.

### 2.3. Biomolecules vs. LMW-Inhibitors

The breakthrough appearance of the first LMW PSMA inhibitors proved their superiority in systematic theranostic approaches. The rapid pharmacokinetic profiles of the LMW peptidomimetics, along with their sufficiently high binding affinities and internalisation ratios, proved to be more favourable in PSMA-TRT as compared to other molecular formats. Additionally, besides therapeutic approaches benefiting from LMW inhibitors, diagnostic tools were also improved, with the possibility of employing short-lived isotopes such as ^18^F of ^68^Ga. Furthermore, the radiation burden to healthy tissue decreased with the celerity of clearance, along with a lesser concern regarding severe side reactions after administration and much-reduced costs of production when compared to mAbs; however, limitations of LMW ligands have also been reported, as they show unspecific binding in healthy tissue, i.e., salivary glands and kidneys, a shortcoming that is not present in mAbs. As such, further studies are still needed to finally determine the molecular format with the best balance between efficacy and side effects. At the moment, this seems to strongly depend on the particular clinical scenario.

## 3. Targeted Radionuclide Therapy of PSMA-Positive Prostate Cancer

### 3.1. Beta Minus (β^-^)-Based Targeted Radionuclide Therapy

**Figure 3:**
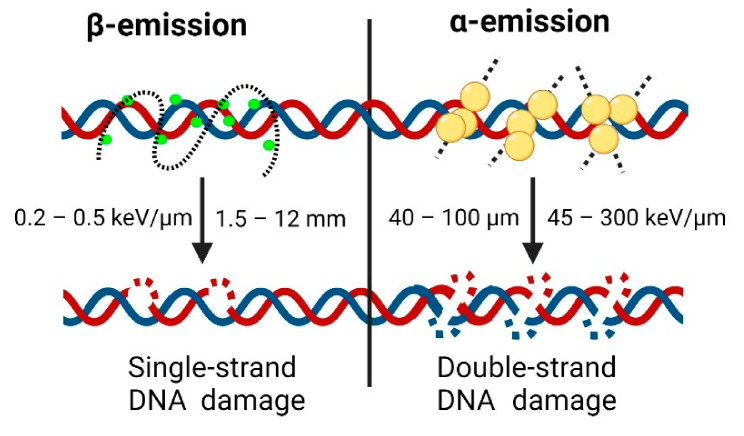
Schematic drawing showing differences between β^-^-and α-emission. The β^-^-emission is less focused, with a longer range and lower LET, leading to single-strand DNA damage. The α-emission is highly energetic, focused and short in range, causing double-strand DNA breaks.

Beta minus emission is the process by which a radioactive nuclide emits an electron (β^-^-particle). After this emission, the daughter nucleus has one more proton and one less neutron. The β^-^-particles have a relatively low linear energy transfer (LET) range of around 0.2 to 0.5 keV/μm [111]. This low LET range means β^-^-particles are sometimes not powerful enough to fully eradicate the tumours, as they are occasionally not capable of causing comprehensive irreversible DNA damage (Figure 3) [112]. Additionally, β^-^-particles have a relatively long range in tissue (from 1 to 10 mm), which can result in collisions and consequent damage to healthy tissue surrounding the cancerous lesion; however, these intrinsic properties lead to what is known as the ‘crossfire effect’. The effect results in the destruction of not only the tumour, but also healthy stroma in the nearby vicinity, leaving the cancerous cells without a viable environment to survive. Consequently, this allows the treatment of macroscopic, cluster-like metastases without the radiopharmaceutical having to be linked to (or in the direct vicinity of) each cancerous cell [113].

#### 3.1.1. β-Emitting Radionuclides for TRT

Only a limited number of the nuclides making up the periodic table are classified as β^-^-emitters. Within this category, there is an even more select list that represents β^-^-emitters suitable for nuclear medicine and TRT. The feasibility of a β^-^-emitter for TRT is defined by a series of basic prerequisites—having a sufficient but not overly long half-life, adequate β^-^-emission energy with a constrained range, availability of the radionuclide and ease of conjugation with the respective pharmaceutical. In general, the majority of β^-^-emitters employed today are radiometals, which are usually conjugated to molecules via chelators such as DOTA, DOTAGA or NOTA.

All of the radionuclides listed in Table 1 have been employed in the treatment of mCRPC, either in preclinical or clinical settings. ^89^Sr has been used as a ‘calcium mimic’ in the management of pain caused by late-stage osseous metastases and is generally not suitable for TRT applications due to its very long half-life and lack of practical chelators [114].

Copper-67 has recently emerged as an especially interesting therapeutic radionuclide due to its favourable half-life, β^-^-emission energies, range and applicability in simultaneous therapy or SPECT, reducing the complexity in the diagnostic therapy timeline [115]. Moreover, the existence of ^64^Cu, a positron emitter, permits its application as an ‘authentic theranostic’ pair.

In the case of ^90^Y, its half-life is ideal for systematic therapeutic approaches, although the main downsides with the radionuclide are its significantly high emission energy and long range, which often lead to excessively unspecific bystander healthy tissue damage [117]. In contrast, ^177^Lu has so far been the most widely applied radionuclide for β^-^-TRT. In addition, its half-life is adequate for allowing sufficient accumulation in the first days after administration, while decaying near the cancerous lesions. Furthermore, its range and emission energy are not excessive and generally limit the exposure of healthy stroma to radiation [118].

As for ^131^I, which is one of the few non-metallic β^-^-emitters, its properties are generally suitable for its application in TRT. Moreover, it emits gamma rays, which are energetic enough for SPECT imaging. Nevertheless, iodine needs to be covalently bound to the pharmaceutical, which can pose challenges in the synthetic pathway of the radiopharmaceutical. Additionally, iodine is known to accumulate in tissues such as thyroids, often making them a dose-limiting factor in ^131^I-based TRT [119].

Finally, terbium-161 is an experimental radiometal that is promising for its application in TRT. ^161^Tb emits β^-^ particles, along with Auger and conversion electrons. This particularity of ^161^Tb makes it deliver higher radiation doses directly to the cancerous tissue and gives it the ability to cause double-strand DNA damage, which can be more effective in clearing cancerous tissue [120].

Taking into consideration the various available radionuclides, it is apparent that the applicability of β^-^-TRT in PSMA(+) PC depends on the nature of the tumours, stage of disease and the administered dose or radionuclide, which should allow for a dose high enough to destroy tumours but constrained to limit unspecific healthy tissue damage.

#### 3.1.2. Preclinical PSMA β^-^-TRT

From the beginning of the early development of PSMA-targeting entities, researchers have sought to apply β^-^-TRT to treat hormone-refractory PSMA(+) PC.

In this regard, the first preclinical evaluation of β^-^-TRT was performed using the intracellular mAb 7E11. The mAb was mainly developed and evaluated by employing its ^111^In-labelled version, even though its ^90^Y version (^90^Y-CYT-356) was characterised in preclinical settings before the first-in-human translation [121]. Nevertheless, as previously noted, the main burden in 7E11-based approaches is the intracellular binding of the mAb; therefore, scientists applied the extracellular-binding mAb, J591, in β^-^-TRT of PC. For this purpose, a variety of radionuclides, ranging from ^131^I to ^90^Y and finally ^177^Lu, were assessed in preclinical studies [20,22,122,123]. The best candidate for clinical translation was found to be the ^90^Y- and ^177^Lu-labelled huJ591. The radiolabelled mAbs showed a significant dose tolerability along with an antitumour response, namely up to 90% reduction in mean tumour volume and 2–3-fold increases in median survival times after a single 3.7–7.4 MBq dose.

As previously discussed, PSMA-targeting LMW ligands have been shown to have the ideal pharmacokinetic properties for TRT, namely specificity, efficient penetration, high uptake in PSMA(+) cancerous cells and rapid blood clearance. Extensive preclinical therapeutic evaluations in mouse xenografts were not initially carried out for many of the LMW TRT applications, with the main reason being that the vast majority of LMW ligands suited the definition of a theranostic agent allowing preclinical evaluations with the imaging counterparts of the inhibitors. Such is the case for MIP-1095, which was only evaluated preclinically as the ^123^I-labelled version before first-in-human studies [80]. In contrast, PSMA-617 was tested in a LNCaP xenograft as the gallium-68 conjugate, although its biodistribution was also evaluated with the ^177^Lu-labelled variant. In both cases, the radiopharmaceutical showed specific uptake in PSMA(+) cancerous tissue, sufficient tumour uptake up to 24 h p.i. (10.58 ± 4.50%ID/g) and rapid renal clearance [92,95]. PSMA I&T, developed by Weineisen et al. and evaluated in a mouse LNCaP xenograft, also showed specific PSMA-mediated uptake 1 h p.i., both as the ^68^Ga (4.95 ± 1.57%ID/g) and ^177^Lu (7.96 ± 1.76%ID/g)-labelled versions [93].

In 2019, Müller et al. reported a terbium-161 variant of PSMA-617. The radiopharmaceutical was tested in PSMA(+) PC-3 PIP tumour-bearing mice, showing the same pharmacokinetic profile as [^177^Lu]Lu-PSMA-617. Interestingly, [^161^Tb]Tb-PSMA-617 was found to have an enhanced therapeutic effect on PSMA(+) PC-3 PIP tumours with higher survival times among the mice when compared to its ^177^Lu counterpart (65 vs. 36 days). The authors attributed the enhancement of therapy to the ^161^Tb properties, as it does not only emit β^-^-particles, but also Auger and conversion electrons, which might increase the absorbed dose and thereby cause damage to the tumours [124].

More recently, McInnes et al. developed a bivalent LMW PSMA inhibitor named CuSarbisPSMA. The ligand was radiolabelled with the theranostic pair ^64^Cu/^67^Cu. Firstly, PET imaging studies were carried out in a mouse LNCaP xenograft with [^64^Cu]CuSarbisPSMA to assess the biodistribution and pharmacokinetic profile of the radiopharmaceutical [125]. Thereafter, its therapeutic efficacy was evaluated with [^67^Cu]CuSarbisPSMA in a head-to-head comparison with [^177^Lu]Lu-PSMA-617. The results showed dose-dependent inhibition of tumour growth, which was comparable between the two radiopharmaceuticals at 13 days p.i. (30 MBq or 2 × 15 MBq) in a LNCaP mouse xenograft [126].

In general, preclinical evaluations, both with therapeutic and diagnostic radionuclides, serve as proof-of-concept for clinical translation of PSMA-targeting radioligands for β^-^-TRT of recurrent PC.

#### 3.1.3. Clinical PSMA β^-^-TRT

As already mentioned, considering the intracellular binding properties of 7E11, Deb et al. reported in 1996 one of the first approaches for β^-^-TRT of PSMA(+) PC with a DTPA-conjugated version of the mAb radiolabelled with ^90^Y. The radiopharmaceutical was administered to 12 mCRPC patients and the study concluded that “no patient attained a complete or partial response based on PSA or radiological criteria” [127].

In the same way as in the preclinical tests, efforts shifted towards the extracellular-binding mAb J591. The ^90^Y- and ^177^Lu-labelled versions of the mAb were used in several clinical settings. In a significant number of studies, various doses of the β^-^-radiolabelled mAb were administered. Initial studies found that both [^90^Y]Y- and [^177^Lu]Lu-J591-based therapy could selectively target mCRPC tumourous lesions; however, PSA decreases ≥ 50% were initially limited to ~20% of patients and dose-limiting reversible bone marrow suppression was also reported [25,128,129]. The same researchers applied [^177^Lu]Lu-J591 in a phase II clinical trial, which showed similar results to the phase I studies and an overall progression-free survival period of 82 days between the treated patients [130]. Furthermore, dose fractionation studies were also performed, which proved the feasibility of increasing the dosing while controlling excessive myelotoxicity [131]. Moreover, [^177^Lu]Lu-J591 combined with the chemotherapeutical agent docetaxel has been shown to generally improve response with PSA level decreases > 50% in 73% of the treated mCRPC patients [132].

Nevertheless, the development of LMW PSMA ligands shaped the clinical approaches in the treatment of mCRPC. In this manner, the first developed and preclinically evaluated MIP-1095 was radiolabelled with the therapeutic β^-^-emitter iodine-131. [^131^I]I-MIP-1095 was administered to 28 mCRPC patients in a single-dose (mean activity: 4.8 GBq) study that showed good uptake in tumourous lesions. Here, 61% of the patients showed decreases in PSA levels > 50%, while symptoms such as bone pain were diminished in 85% of the patients [77]. Nevertheless, further studies with repeated doses of [^131^I]I-MIP-1095 did not show improvements of the therapeutic effects caused by multiple dosing, often not completely eradicating the tumours and showing disease relapse in some cases. Additionally, the repeated administration of the radiopharmaceutical increased the dose-limiting uptake in the salivary glands, liver and kidneys [133]. Despite [^131^I]I-MIP-1095 limitations, the clinical application still demonstrated the potential of LMW ligands in the clinical management of recurrent PC.

Furthermore, in 2015, German Cancer Research Centre scientists applied PSMA-617 in a first-in-human study. Owing to the excellent targeting properties and pharmacokinetic profile of this radiopharmaceutical, the administration of a cumulative activity of 7.4 GBq (2 doses) of [^177^Lu]Lu-PSMA-617 to a single mCRPC patient showed positive results, with an unprecedented radiological response and PSA levels dropping from 38.0 to 4.6 ng/mL [134]. Interestingly, in parallel, researchers from the Technical University of Munich developed PSMA I&T and also applied it in a first-in-human single-dose study as the ^177^Lu-radiolabelled version administered to two (5.7 and 8.0 GBq) mCRPC patients [93]. [^177^Lu]Lu-PSMA I&T showed specific tumour uptake and revealed partial remission of PSMA(+) tumours accompanied by PSA decline (from 40.2 ng/mL to 0.7 ng/mL for one patient). Both PSMA-617 and PSMA I&T performed significantly better than MIP-1095 and showed less side effects and less unspecific uptake. Consequently, both radiopharmaceuticals were translated in larger clinical cohorts. Nevertheless, clinical development has been much more extensive for PSMA-617. Table 2 summarises some of the most relevant retrospective data and clinical studies employing [^177^Lu]Lu-PSMA-617 over the last 5 years.

In general lines, the clinical studies treating hormone-refractory PC with [^177^Lu]Lu-PSMA-617 demonstrate that approximately 75% of patients show response regarding PSA level decline. Additionally, PSA decreases > 50% were observed in ~55% of the treated men. Nevertheless, the data from the clinical studies are vastly heterogeneous, as the starting point of therapy, disease stage and localisation of lesions play important roles in the outcome. Another factor to weigh in assessing the effectivity of treatment is the injected dose and the number of cycles, which were also heterogenous across the reported studies. Furthermore, the treatment is well tolerated, as toxicities in vital organs have generally not been observed and severe haematotoxicity has been reported in very few cases. Unfortunately, salivary gland uptake might be a dose-limiting factor in systematic, multidose treatment regimens due to specific and unspecific uptake of PSMA-targeting LMW ligands [146,147,148].

Currently ongoing β^-^-TRT clinical trials for mCRPC patients are evaluating a variety of radiopharmaceuticals. A modified albumin-binding version of PSMA-617, [^177^Lu]Lu-PSMA-EB-617, is currently being tested in a phase I clinical trial [149]. A phase II study is evaluating the outcome of patients treated with the antibody [^177^Lu]Lu-huJ591 co-administered with the steroid ketoconazole [150]. Another ongoing phase II clinical trial based on [^177^Lu]Lu-PSMA-617 is comparing this agent head-to-head with the conventional treatment option cabazitaxel [151]. Finally, a large-scale phase III clinical study (SPLASH) is currently recruiting patients to evaluate the efficacy and safety of [^177^Lu]Lu-PSMA I&T in mCRPC patients following androgen-based therapy [152].

Independently of the ongoing clinical studies, in general lines, [^177^Lu]Lu-PSMA-617 has been demonstrated to be a viable therapeutic option for mCRPC, with acceptable response rates and manageable side effects. In a phase II clinical trial conducted at the Peter MacCallum Cancer Centre in Australia, the efficacy, safety and quality of life of patients with mCRPC were analysed, showing PSA decreases of more than 50% in 57% of patients and PSA decreases of more than 80% in 43% of patients. No grade 3/4 non-haematological and no renal toxicities were observed. An exceptional response case was also part of this phase II study. A patient with widespread mCRPC and a PSA of >900 had a complete radiologic response accompanied by a PSA decrease of 99% following treatment with [^177^Lu]Lu-PSMA-617 [141].

The most advanced clinical trial that [^177^Lu]Lu-PSMA-617 has undergone is a recently concluded phase III trial (VISION) with 831 patients. The recently published data indicate that [^177^Lu]Lu-PSMA-617 significantly improves overall survival in men with mCRPC, reducing the risk of death by 38% and reducing radiographic disease progression by 60%, bringing the radiopharmaceutical close to FDA approval [145].

### 3.2. Alpha (α)-Based Targeted Radionuclide Therapy

Alpha (α) emission is the process by which a radionuclide emits an α particle, which is identical to a helium nucleus (2 protons + 2 neutrons). In contrast to β-emitting radionuclides, α-emitters have extremely high particle energy (~7500 keV). This amount of energy is capable of causing double-strand DNA breaks, which generally render the cancerous cells unable to repair (Figure 3) [153]. Moreover, the range of those particles in tissue is extremely short (~80 μm), preventing damage to healthy stroma neighbouring the cancerous cells. For these reasons, α-emitters have been postulated as ideal radionuclides for the treatment of cancer [154,155,156,157]. In particular, the treatment regimen for disseminated and poorly differentiated metastatic disease, e.g., PSMA(+) mCRPC, might highly benefit from targeted α radionuclide therapy (TAT) [158]. Recently, extensive efforts have been made to review in detail α-emitter-based radiotherapeutic approaches for PC [159]. This section focuses on a general outline of the main preclinical and clinical milestones for PSMA TAT.

#### 3.2.1. α-Emitting Radionuclides for TRT

Homologous to β-emitters, a limited number of nuclides classify as α-emitters. Furthermore, the applicability of α-emitters in nuclear medicine is limited to the properties of the radionuclide. Requisites such as half-life, number of α-decays per nuclide as well as side non-α decay influence the feasibility of using certain α-emitters in systematic approaches.

In respect to Table 3, all the listed α-emitters have been employed in preclinical or clinical settings for the management of PC. In fact, ^223^RaCl_2_ (Xofigo^®^) represents the only α-emitting radiopharmaceutical approved by the FDA for clinical use. Equivalent to ^89^Sr, ^223^Ra is used to non-specifically target mCRPC bone metastases by mimicking calcium and being absorbed into the bones, where it eliminates invasive cancerous tissue, decreasing patient pain and elongating their survival [160].

Bismuth-213 was the first α-emitter conjugated with anti-PSMA mAbs for its application in TAT. ^213^Bi is a mixed emitter, giving both α- and β^-^-particles. Aside from not being a pure α-emitter, it also has the limitation of a relatively short half-life of 45 min, which does not match the typical pharmacokinetic profiles of the majority of PSMA-targeting radiopharmaceuticals [161].

Actinium-225 was the first α-emitting radionuclide to be conjugated with the ‘gold-standard’ PSMA-617 for its application in TAT. Although some studies have shown impressive efficacy in single cases of PSMA-TAT, actinium-225 is far from being a routine radionuclide for systematic approaches. As with other α-emitters, the radionuclide exhibits certain drawbacks that are mainly related to its decay properties. Multiple α-active daughter atoms are able to recoil from the parental radiopharmaceutical, which potentially results in high toxicity in cumulative tissues. Furthermore, ^225^Ac is a very scarce element that cannot be easily produced in a cyclotron [162]. In addition, its decay chain releases detectable photons only after its first decay (t_1/2_ = 10 days), making its detection difficult and raising radiation protection concerns for routine production.

Astatine-211 (^211^At) has so far only been employed in PC preclinical settings. The main drawbacks in the application of ^211^At in systematic approaches are two-fold. Firstly, the capacity for producing astatine is not widespread, as roughly 30 institutions have access to it and its production is often not cost-effective [163]. Secondly, and most importantly, the stability of carbon–astatide bonds in vivo has been discussed and shown not to be as strong as its closest element, iodine. Even though the mechanisms by which astatine is detached from the covalent bond in vivo have still not been elucidated, several hypotheses exist, including oxidative dehalogenation by reactive oxygen species in the body [157,164]. Nevertheless, it remains an extremely promising radionuclide for TAT due to its very favourable decay properties—one α particle per decay without any additional α-emitting daughters or side β-emissions [163]. Additionally, its short-lived daughters emit secondary low-energy X- and γ-rays, which might not be ideal for practical imaging purposes but still present significant safety advantages [165,166].

Lead-212 (^212^Pb) is not an α-emitter per se, but a generator of in situ α-decay. It emits a β^-^-particle to give ^212^Bi, which then releases a helium nucleus, as well as a β^-^-particle to start a series of concomitant decays that end in ^208^Pb. Even though the staggered decay of ^212^Pb is not ideal for the stability, efficacy and biodistribution of the radioactivity, it has the very interesting feature of having a ‘real theranostic pair’. As another isotope of lead, ^208^Pb can be employed for imaging via SPECT [167].

Thorium-227 is a relatively newly developed radionuclide for TAT. Its physical half-life of 18 days allows for viable applications in clinical routines, although its decay properties pose a challenge as it gives up to five α-particles until it reaches the stable ^207^Pb. Homologous to ^225^Ac, the multistep α-decay raises concerns regarding recoiled daughter atoms and accumulation in non-target tissue. ^227^Th-based TAT is still in its early developmental stage and more data are needed to give a solid assessment of its feasibility [162].

Finally, terbium-149 is a recently developed radionuclide for TAT applications. Its short half-life (4.1 h) reduces the radiation burden to non-target tissue and is perfectly suited for conjugation with fast-clearing LMW ligands. Moreover, the emission of a positron in its decay chain allows for PET imaging studies employing the same ligand and identical radionuclide [168]. Nevertheless, the availability of the radionuclide is still very limited for practical clinical applications.

#### 3.2.2. Preclinical PSMA TAT

Consequently, ever since the discovery of the first PSMA-binding mAbs, research has been focused on TAT of mCRPC. The first reported study of TAT of PSMA(+) PC was a preclinical evaluation of ^213^Bi-labelled J591 [169]. In this evaluation, McDevitt et al. tested the therapeutic effect of the mAb in a mouse LNCaP xenograft. The study concluded that the administration of ~3.5 MBq of [^213^Bi]Bi-J591 significantly prolonged the overall survival of LNCaP xenograft mice (54 days for treated group, 33 for control and 31 for non-treated). Furthermore, PSA levels in treated mice were also shown to be significantly decreased after TAT treatment (28 ± 22% ng/mL for the treated group vs. 104 ± 54 ng/mL for the untreated mice) [169]. Additional preclinical studies in mice further confirmed the antitumour activity of [^213^Bi]Bi-J591 [170]. Nevertheless, even given its apparent effectivity in preclinical settings, the ^213^Bi-labelled mAb never progressed into human trials.

After the initial TAT efforts, it was not until 2009 that Wilbur et al. reported another preclinical TAT of PSMA(+) PC. In this case, the mAb 107-1A4 was modified with a closodecaborate for its labelling with the pure α-emitter astatine-211 (^211^At). The treatment was tested in mice implanted with C4-2B cells into the tibia, a situation that simulates bone micrometastases [171,172]. The study showed that a single dose of 370 kBq of [^211^At]At-107-1A4 significantly reduced PSA levels in blood when compared to control groups [172]. Additionally, Wilbur and colleagues further derivatised the mAb 107-1A4 into a Fab’. Following the same strategy as with the mAb, the Fab’ was conjugated with closoborate and labelled with ^211^At. In this instance, only the biodistribution in a LNCaP mouse xenograft was assessed. The [^211^At]At-Fab’ showed an accelerated pharmacokinetic profile when compared to its parental mAb, with good tumour enrichment up to 25 ± 13% ID/g after 24 h but a noticeable kidney accumulation of 40 ± 16% ID/g at the rather late time point of 24 h p.i. [173].

Kiess et al. employed the same radionuclide in further preclinical studies. They reported the first LMW urea-based radiopharmaceutical to be tested in preclinical settings for TAT of PC [78]. The compound, [^211^At]At-DCAtBzL, was administered to mice bearing PC-3 PIP PSMA(+) micrometastases. The evaluation showed satisfactory tumour uptakes 1 h p.i. (20 ± 5% ID/g) and effective antitumour properties, although also severe toxicity. The radiopharmaceutical accumulated in the renal proximal tubes, most likely due to in vivo de-astatination or due to the known high PSMA expression in rodent proximal tubes, which resulted in severe nephropathy [78]. Most recently, the same group of researchers reported the synthesis and preclinical evaluation of a second generation of ^211^At-bearing compounds. In this case, the lead compound, known as [^211^At]At-VK-02-90-Lu, showed in vitro an uptake of 13.4 ± 0.5% 4 h p.i. in PSMA-expressing PC-3 PIP cells. Its evaluation in mouse PC-3 PIP xenografts showed even better uptake (30.6 ± 4.8% ID/g) 1 h p.i., with satisfactory kidney clearance over 24 h and a significant increase in overall survival (dose > 1.48 MBq/mouse) for the treated mice when compared to control groups [174].

Umbricht et al. showed in a preclinical study the feasibility of employing terbium-149 in conjugation with PSMA-617. In an ‘α-PET’ study, [^149^Tb]Tb-PSMA-617 was administered to mice bearing PSMA(+) PC-3 PIP tumours. The administration of 2 doses of 3 MBq each significantly delayed tumour growth. Moreover, the administration of 5 MBq of [^149^Tb]Tb-PSMA-617 also allowed for PET imaging studies. Additionally, the substitution of ^177^Lu for ^149^Tb in PSMA-617 did not have an effect on the biodistribution of the radiopharmaceutical [175].

Further development of a LMW-ligand-based TAT led to the application of the ^212^Pb/^212^Bi tandem into PSMA-617-derived structures. In this sense, Dos Santos and co-workers employed ^212^Pb in the ligands CA009 and CA012 [176]. Both structures were directly derived from PSMA-617, with a substitution of the DOTA chelator for better choices for lead—DO3AM and p-SCN-Bn-TCMC. Both radiopharmaceuticals showed satisfactory biodistribution profiles, especially CA012, with LNCaP-tumour accumulation at 1 h p.i. comparable to PSMA-617 (8.4 ± 3.7% ID/g) and a remarkably lower kidney uptake than PSMA-617 (5.1 ± 2.5% ID/g vs. 113.3 ± 24.4% ID/g). Other similar efforts were reported by Stenberg et al. [177] for the development and evaluation of NG001, the same structure as CA009, which exhibited a very fast pharmacokinetic profile that also outperformed current state-of-the-art PSMA-617 in kidney uptake at 2 h p.i. (21.07 ± 10.33% ID/g vs. 52.82 ± 26.62% ID/g). Finally, Banerjee et al. reported a series of three compounds for their application in ^212^Pb PSMA TAT. Nevertheless, only one of the radioligands, [^212^Pb]Pb-L2, was selected for preclinical testing. It was shown to improve survival times of PSMA(+) PC-3 PIP mouse xenografts, as well as dose-limiting kidney uptake [178].

#### 3.2.3. Clinical PSMA TAT

The discovery and clinical implementation of [^177^Lu]Lu-PSMA-617 was the most relevant milestone in the development of PSMA-TRT, showing great promise in the treatment of mCRPC and currently being on the verge of FDA approval [179]; however, PSMA-617 still has drawbacks. Recent studies have shown that approximately 40% of patients that received [^177^Lu]Lu-PSMA-617 did not respond to the treatment [137,144]. This might be attributed to the intrinsic nature of β^-^-emitting radionuclides, which are potentially not ideal for the treatment of small and disseminated tumours due to their long range in tissue and relatively low energy. In contrast, α-emitters are more effective in treating tumours due to their physical properties (short range and high LET). In this context, Kratochwil et al. reported a variation of PSMA-617 labelled with actinium-225, [^225^Ac]Ac-PSMA-617, as a salvage therapy for patients either non-respondent to or not eligible for [^177^Lu]Lu-PSMA-617 [180]. The administration of [^225^Ac]Ac-PSMA-617 to 2 patients in a first-in-human study showed promising antitumour effects, clearing the metastatic burden of the disease and reducing PSA levels to non-detectable thresholds. In a further clinical study, the efficacy of [^225^Ac]Ac-PSMA-617 was assessed in a 40 patient cohort [181]. The results showed decreases of PSA levels greater than 50% in 63% of the patients, increasing the overall survival time over the median value for mCRPC patients. Remarkably, 13% of the patients showed disease remission for more than 2 years after treatment administration. Nevertheless, the dosing and successful therapeutic cycles of the radiopharmaceutical were heavily influenced by salivary gland uptake, as irreversible xerostomia was a common side effect within the cohort of treated patients [181]. Other clinical studies carried out by different researchers or institutions employing [^225^Ac]Ac-PSMA-617 had similar outcomes. A significant percentage of patients showed disease remission, pronounced decrease of PSA levels and extended overall survival times, although also noticeable dose-limiting xerostomia and some blood disorders, as well as kidney toxicity [155,182,183,184,185].

It is important to note that there are ongoing clinical trials employing mAbs for TAT of PC. For instance, the aforementioned mAb J591, in combination with ^225^Ac, is being used in three different clinical trials [186,187,188].

More interestingly, Bayer recently developed a PSMA-targeted thorium conjugate (PSMA-TTC), which comprises a mAb conjugated with 3,2-HOPO, a molecule capable of chelating the α-emitter ^227^Th. In preclinical testing, the [^227^Th]Th-PSMA-TTC showed promising results with clear antitumour efficacy in various murine PC models [189,190]. As a consequence, the radiopharmaceutical is currently being employed in an ongoing phase I clinical trial, with a sample size of around 150 patients [191].

Overall, these investigations demonstrate the potential of α-radiation to overcome the intrinsic limitations of β-emitters, especially with regard to widespread and poorly differentiated disease, in systematic approaches for treatment of PSMA(+) PC. Therefore, the future of PSMA TRT will be inevitably influenced by the developments in α-emitters and how these can be effectively combined with PSMA-targeting pharmaceuticals without excessive prohibitive side effects [162].

### 3.3. Enhancement of PSMA-Targeted Radionuclide Therapy

**Figure 4:**
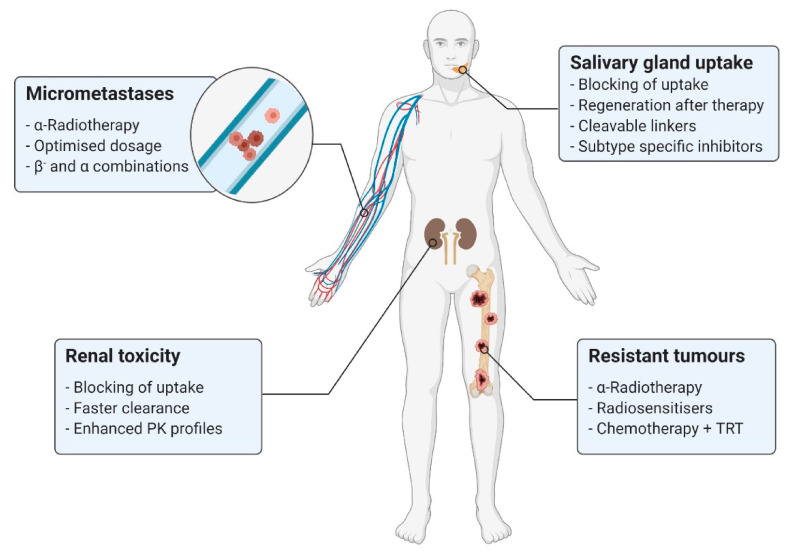
The main challenges associated with clinical PSMA-TRT and possible approaches to overcome these limitations.

In addition to the previously discussed therapeutic effects, improvements based on structural modifications of the pharmaceuticals and combined therapeutic approaches represent other options for enhancing the TRT of prostate cancer. Due to the continued existence of clinical challenges (Figure 4) during PSMA-TRT, such as insufficient therapeutic success or severe side effects, new treatment strategies might result in significant advantages for patients.

#### 3.3.1. Combinatorial PSMA-Targeted Therapy

Combinations of radio- and chemotherapy have already demonstrated high potential when compared to single therapies in clinical trials [192]. Due to the relative novelty of PSMA-TRT, only a few in-depth combinatorial studies have been conducted.

One approach to enhance the effectiveness of TRT is the use of chemical sensitisers to increase the radiosensitivity of the tumour. Cytotoxic drugs increase the vulnerability to DNA damage in cancer cells, resulting in better tumour clearance in TRT. Although most of these studies were performed in an external beam radiotherapy setting, the results demonstrate the potential of this combined approach [193].

The preclinical study by Tesson et al. showed the effects of the therapy with [^131^I]I-MIP-1095 in combination with different chemotherapeutics (olaparib, topotecan, bortezomib, nutlin-3 and disulfiram) on the tumour growth in LNCaP xenograft-bearing mice [194]. All agents significantly reduced [^131^I]I-MIP-1095-induced tumourous spheroid growth. In another study, the joint effects of [^177^Lu]Lu-PSMA-617 and the radiosensitising substance idronoxil (NOX66) were investigated in men with mCRPC [195,196]. The trial included thirty-two men receiving up to six cycles of [^177^Lu]Lu-PSMA-617 (7.5 GBq) on day 1, with escalating doses of NOX66 on days 1–10 of a 6-week cycle [195]. Overall, 91% of subjects had detectable PSA responses, with a median overall survival rate of 17.1 months [195].

The most studied combinatorial approach remains the tandem application of docetaxel as a chemotherapeutic agent. Targeting the microtubular network in cells, treatment with docetaxel results in the desired radiosensitisation of prostate cancer cells [197]. The first approaches to combine docetaxel with TRT were performed by Kelly et al. with the labelled monoclonal antibody [^177^Lu]Lu-hu3S193, showing significantly improved efficacy in in vivo studies [198]. Similar improvements were shown in a phase I study of docetaxel with the ^177^Lu-labeled PSMA-targeting antibody J591 [199]. This combination appeared to have significant antitumour activity after application of docetaxel (75 mg/m^2^) every 3 weeks combined with 2 fractionated doses of [^177^Lu]Lu-J591 (1.48 GBq/m^3^ up to 2.96 GBq/m^3^). Eighty-three percent (83%) of patients showed PSA response, with a median overall survival rate of 18.4 months. Addressing mCRPC, the UpFrontPSMA Trial is investigating in a randomised phase 2 study the therapeutic options for sequential [^177^Lu]Lu-PSMA-617 and docetaxel treatment vs. docetaxel alone [200]. Furthermore, Maharaj et al. described favourable effects of ^177^Lu-PSMA therapy in combination with docetaxel in a case report of a patient with mCRPC [201].

Clearly, the possibilities for combinational therapeutic approaches are not exhausted at this point and require further research. For instance, combined therapies of TRT with immunotherapy, external beam radiotherapy or chemotherapeutics could lead to better outcomes for PC patients.

#### 3.3.2. Side Effect Minimisation

The application of radiopharmaceuticals involves the challenge of administering highly cytotoxic radioactive nuclides. These can lead to severe damage to healthy tissue if undesired accumulation occurs. The cumulative off-target organs of PSMA-targeting pharmaceuticals are mainly the kidneys and salivary glands; therefore, the current efforts are mainly focused on reducing radiopharmaceutical uptake in these off-target tissues.

Despite its tremendous potential, PSMA-TRT must balance between prolongation of survival and disease-related symptoms versus the direct side effects of the treatment. Xerostomia is perhaps the most frequent and potentially debilitating of these conditions. The symptoms depend on the absorbed dose and the employed isotope, although they are especially relevant for ^225^Ac-LMW-based approaches. In contrast to small molecules, accumulation in salivary glands does not occur during therapy with radiolabelled PSMA-targeting antibodies [30]. Some suggested explanations for the disparity are differences in molecular weight and the specific ionic charge of PSMA radioligands [202]. The low accumulation of antibodies in the salivary glands indicates, at least partially, non-specific salivary gland uptake pathways [59]. The results from the studies by Rupp et al. and Tönnesmann et al. on [^68^Ga]Ga-PSMA-11 and [^177^Lu]Lu-PSMA-617, respectively, further confirmed this thesis [145,147].

Data from different studies after therapy with [^177^Lu]Lu-PSMA-617 revealed only mild to moderate xerostomia [138,203]; however, in a study performed by Kratochwil et al. applying [^225^Ac]Ac-PSMA-617, severe xerostomia occurred frequently and was reported to be dose-limiting [181]. To address this problem, various strategies were investigated in clinical trials in order to protect the salivary glands.

In a first approach, external cooling of the salivary glands was expected to reduce the PSMA inhibitor uptake due to vasoconstriction [204]. Although ice packs were affixed over one parotid gland one hour before and four hours after application, no differences could be found in side-by-side comparisons.

In order to supress the metabolism of the gland, Baum et al. injected botulinum toxin in the parenchyma to reduce off-target uptake [205]. In a first-in-human study, a decreased uptake in the pretreated parotid gland of up to 64% was observed when compared to baseline.

Inspired by data on thyroid cancer patients with radioiodine-induced sialadenitis, sialendoscopy represents another option of treatment [206]. In a study of 11 patients, Rathke et al. investigated the effects of saline irrigation and steroid injection in the salivary glands before or after every cycle of [^225^Ac]Ac-PSMA-617. Although beneficial effects on the salivary gland functions were observed, chronic xerostomia still occurred after multiple cycles of ^225^Ac-TAT.

Attempts at blocking the salivary glands with non-radiolabelled PSMA inhibitors such as 2-PMPA [207], TrisPOC-2PMPA [208] or PSMA-11 [209] were also investigated to prevent specific binding. The results showed significant reductions of tracer uptake in the salivary glands and the kidneys, although at the cost of diminished uptake within the tumour. The recent studies by Harsini et al. on the effects of monosodium glutamate on PSMA tracer uptake resulted in similar effects as blocking with the cold compounds [210]. Furthermore, orally administered folic acid may also reduce the absorbed dose to the salivary glands, as recently shown by Paganelli et al. [211]. Finally, Sarnelli et al. investigated the potential of administering polyglutamate as a salivary gland protector combined with mannitol to reduce kidney uptake [212]. Unfortunately, the clinical results showed no significant effects on the accumulation of PSMA-targeting inhibitors in both organs.

In conclusion, the preventive strategies seem to be limited, with a still existing urgent need for further developments. A way to bypass the problem might be the induction of regeneration of the salivary glands by application of stem cells after radiation damage [213]. Nevertheless, research is warranted in this area, especially as TAT approaches gain relevance, in order to enhance patient care and decrease after-treatment morbidity.

## 4. Conclusions and Future Outlook

In recent years, PSMA-TRT has arisen as a feasible alternative to conventional treatments in the field of mCRPC therapy. This rapid success story has had a huge impact on the field of radiopharmaceutical research and encouraged major pharmaceutical industries to intensify their efforts in the field of nuclear medicine.

In addition, research activities have yielded a wide range of suitable PSMA-targeting radiopharmaceuticals that are available today, ranging from mAbs and mAb-derived structures to LMW agents. Nevertheless, after clinical translation, LMW inhibitors of PSMA seem to represent a favourable molecular format for TRT and TAT. In particular, current phase III results of [^177^Lu]Lu-PSMA-617 impressively demonstrate the great potential of LMW-based TRT for mCRPC, amounting to the best prerequisites for regulatory approval. Moreover, recent FDA authorisations for [^68^Ga]Ga-PSMA-11 and [^18^F]F-DCFPyL further support the approval for PSMA-617.

Additionally, studies with α-emitters conjugated to PSMA-targeted ligands have gained significant impact over recent years, with encouraging results related to their efficacy, especially in β^-^-resisting lesions; however, accumulation of the inhibitors in the kidneys and high uptake rates into the salivary glands remain the most challenging limitations in the field of PSMA-TRT.

In conclusion, the favourable properties of LMW inhibitors, along with the proven performance of PSMA-617, warrant a focus on these agents in PC-related research. Furthermore, current challenges such as undesired uptake in non-target organs, disease relapse due to micrometastases, tumour heterogeneity and resistance will need to be addressed through the development of combined therapeutic approaches, as well as the introduction of the long-promised α-emitters in systematic clinical settings.

## Figures and Tables

**Table 1:** The β^-^-emitters used in nuclear medicine, along with their basic properties. Data obtained from [112,116].

Radionuclide	Half-Life	Emission	E_β(max)_/Range (Max)
^67^Cu	61.9 h	β^-^/γ	575 keV/2.1 mm
^89^Sr	50.5 d	β^-^	1491 keV/7.0 mm
^90^Y	64.1 h	β^-^	2284 keV/11.3 mm
^131^I	8.0 d	β^-^/γ	606 keV/2.1 mm
^161^Tb	6.9 d	β^-^/Auger/CE	150 keV/0.1 mm
^177^Lu	6.7 d	β^-^/γ	497 keV/1.8 mm

**Table 2:** The main retrospective analyses and clinical studies carried out with [^177^Lu]Lu-PSMA-617.

Year	Num. of Patients	% with PSA Decline > 50%	Mean Administered Dose per Cycle/s	Authors and References
2015	10	50%	5.6 GBq (1 cycle)	Ahmadzadehfar et al. [135]
2016	24	42%	6.0 GBq (2 cycles)	Ahmadzadehfar et al. [136]
2016	74	31%	5.9 GBq (1 cycle)	Rahbar et al. [137]
2017	99	40%	5.9 GBq (1–4 cycles)	Rahbar et al. [138]
2017	52	44%	6.0 GBq (3–6 cycles)	Ahmadzadehfar et al. [139]
2018	104	33%	6.1 GBq (1–8 cycles)	Rahbar et al. [140]
2018	30	57%	7.5 GBq (2–4 cycles)	Hofman et al. [141]
2019	30	23%	8 GBq (1–6 cycles)	Yordanova et al. [142]
2019	32	38%	6 GBq (2–6 cycles)	Maffey-Steffan et al. [143]
2020	54	58%	7.5 GBq (3 cycles)	Rasul et al. [144]
2021	385	46%	7.4 GBq (4–6 cycles)	Sartor et al. [145]

**Table 3:** α-Emitters employed in mCRPC treatment and their properties. Data extracted from [110,111].

Radionuclide	Half-life	Emission	E_α(max)_/Range(Max)
^149^Tb	4.1 h	α/β+	3.97 MeV/28 µm
^211^At	7.2 h	α	6.79 MeV/60 µm
^212^Pb	10.6 h	β^-^ to α ^212^Bi	6.05 MeV/80 µm
^213^Bi	45.6 min	α/β^-^	8.32 MeV/84 µm
^223^Ra	11.4 d	α	5.64 MeV/45 µm
^225^Ac	10.0 d	α/β^-^	6.83 MeV/61 µm
^227^Th	18.7 d	α	6.14 MeV/100 µm
